# Interest in lifestyle advice at lung cancer screening: Determinants and preferences

**DOI:** 10.1016/j.lungcan.2018.11.036

**Published:** 2019-02

**Authors:** Claire Stevens, Samuel G. Smith, Samantha L Quaife, Charlotte Vrinten, Jo Waller, Rebecca J. Beeken

**Affiliations:** a Department of Behavioural Science and Health, University College London, London WC1E 6BT, UK; b Leeds Institute of Health Sciences, University of Leeds, Leeds, LS2 9NL, UK

**Keywords:** Lung cancer, Cancer screening, Primary prevention, Smoking, Tobacco, Alcohol consumption, Diet, Physical activity, Body weight, Lifestyle risk reduction

## Abstract

**Background:**

Lung cancer screening could be a ‘teachable moment’ for behaviour change. Little is known about how advice about smoking cessation, or other behavioural cancer risk factors, would be received in this setting.

**Methods:**

Using a population-based survey of 459 English adults (current smokers and recent quitters aged 50–75) we assessed willingness to receive lifestyle advice (about smoking, diet, weight, physical activity, alcohol consumption) at lung screening. Additional items assessed whether advice should be provided following abnormal screening results, the potential impact of advice on screening uptake, and preferred timing of advice.

**Results:**

Overall, 64% (n = 292) of participants were willing to receive lifestyle advice at lung screening. A greater proportion of participants were willing to receive advice in a scenario where results required further investigation (83%; p < 0.01). However, 14% indicated the provision of lifestyle advice would make them less willing to attend lung screening. Non-White ethnicity and greater cancer risk factor awareness were associated with willingness to receive advice (p < 0.05). Half of smokers (51%) were willing to receive cessation advice. There was also interest in advice about diet (47%), weight (43%), physical activity (32%), and alcohol consumption (17%) among people not meeting current recommendations for these behaviours. There was a preference for advice to be delivered at the screening appointment (38%, n = 108) over other time-points.

**Conclusions:**

Lung screening may offer an opportunity to provide advice about behavioural cancer risk factors. Future work should consider how to deliver effective interventions in this setting to support behaviour change, without affecting screening uptake.

## Highlights

• We explored willingness to receive advice about lifestyle at lung screening.
• Two thirds of lung screening intenders were willing to receive lifestyle advice.
• They were willing to receive smoking, diet, weight and physical activity advice.
• They preferred advice to be at the screening appointment itself.
• Interest was associated with ethnicity and greater awareness of cancer risk factors.

## 1 Introduction

The European position statement on lung cancer screening recommends low dose computed tomography for high risk populations, with smoking cessation advice offered alongside [1]. The prevalence of tobacco smoking ranges from 10 to 38% between European countries [2]. In England, 17% of adults smoke [3]. However, data from lung screening trials suggests smoking prevalence is likely to be higher among lung screening attendees [[4], [5], [6]]; within the UK Lung Cancer Screening (UKLS) pilot trial, 38.3% of the screening arm were current smokers [4]. Tobacco is the single greatest contributor to cancer burden in the European Union [7]. However, health behaviours tend to cluster, meaning smokers are more likely to engage in other cancer risk behaviours, including increased alcohol consumption and eating an unhealthy diet [8,9]. These modifiable behaviours contribute to many non-communicable diseases including cancer, cardiovascular and respiratory diseases, and diabetes [10]. The lung screening population may therefore stand to benefit considerably from a multiple risk factor approach to behaviour change. Combining cancer screening and behaviour change advice could be cost effective and provide the greatest impact on mortality [[11], [12], [13]], with the addition of smoking cessation interventions alone estimated to improve the cost effectiveness of lung screening by 20–45% [14].

Behaviour change interventions delivered within lung cancer screening trials have focused on smoking cessation, with a recent systematic review showing tentative positive support for their implementation [15,16]. To our knowledge, no research has explored the implementation or acceptability of advice about other cancer risk factors at lung cancer screening. It is therefore important to consider the acceptability of advice about multiple risk factors within this context. Most people appear to be willing to receive lifestyle advice about a range of cancer risk factors at breast, bowel and cervical screening [[17], [18], [19]]. Encouragingly, those with unhealthy behaviours appear to be more willing to receive advice, as do those who are more aware of risk factors for cancer [19]. However, there is concern that providing lifestyle advice at cancer screening may negatively impact uptake [17]. Lung cancer screening uptake is low, particularly among current smokers and socioeconomically deprived populations [20,21]. It is therefore important to explore preferences for advice at lung cancer screening, as well as determinants of interest in that information to avoid exacerbating socioeconomic inequalities further.

Using a nationally representative sample of English adults and hypothetical lung screening scenarios, this research aimed to estimate the proportion of screening intenders interested in receiving lifestyle advice alongside lung cancer screening. We also aimed to identify determinants of interest in lifestyle advice at lung cancer screening, and people’s preferences for the timing and topics of advice.

## 2 Methods

### 2.1 Design

Data were collected using the Smoking Toolkit Study, a monthly cross-sectional survey of smoking trends in England between May-November 2017 (total survey n = 11,899) [22]. Face to face computer-assisted interviews were conducted by market research agency Ipsos MORI as part of an omnibus survey. Ethical approval was granted by the University College London Research Ethics Committee (REF: 5210/002), and informed consent was obtained prior to each interview.

### 2.2 Participants

Random location sampling was used. Quotas were set for sex, age, working status and housing tenure to ensure a nationally representative sample. Participants received questions relating to acceptability of lifestyle advice at a hypothetical lung cancer screening programme based on age and smoking history. Using UK Lung Cancer Screening trial criteria, we included adults aged 50–75 [23]. It was not possible to calculate individuals’ lung cancer risk, so the sample was limited to participants who smoked, or who had quit smoking within the last 5 years. Participants were included in analyses if they stated they would attend lung cancer screening if invited, and were excluded if they had a previous cancer diagnosis.

### 2.3 Measures

Full measures are reported in Supplementary File 1. Data were collected for four sociodemographic variables: age, gender (male / female), ethnicity (White / non-White) and educational attainment as a marker of social position (education to degree level or above / education below degree level).

Participants were provided with a brief description of CT lung screening prior to questions relating to lung screening: ‘A new screening test is being developed to find lung cancer at an early stage. It would use a type of x-ray called a chest CT scan. The scan takes pictures of the lungs which are then checked for the early signs of lung cancer. The screening test would be offered to people who smoke or used to smoke, not people going to their GP with symptoms of lung cancer.’

Intention to attend lung screening was assessed by responses to: ‘If your GP invited you to have a lung cancer screening test as part of an NHS lung cancer screening programme, would you take up the offer?’, dichotomised into yes (Yes, definitely; Yes, probably) and no (No, probably not; No, definitely not).

Lung screening intenders were asked: Would you be willing to receive advice about making healthy lifestyle changes (for example, diet or physical activity) as part of a lung screening programme?’. An additional question assessed interest in lifestyle advice in the event of a screening result which required further investigations: ‘Would you be willing to receive lifestyle advice if your screening result suggested you needed to have further investigations?’. Both responses were categorised into willing (Yes, definitely; Yes, probably), or not willing (No, probably not; No, definitely not; Not sure). A further question asked: ‘If you knew you would receive advice about lifestyle (for example, diet or physical activity) as part of a lung cancer screening programme, would this affect your willingness to attend lung screening?’ (Yes, I would be more willing to attend; Yes, I would be less willing to attend; No, it would not affect my willingness to attend).

Lung screening intenders who were willing to receive lifestyle advice at lung screening were asked about their interest in five topics: *‘If you were to attend lung cancer screening in the future, which of the following, if any, would you be interested in receiving information or advice about?.. How to have a healthy diet / maintain a healthy weight / increase your physical activity / to stop smoking / reduce your alcohol consumption / none of these.’* (Yes /No). One item assessed preferred timing of advice: ‘*When would you prefer to receive lifestyle advice as part of a lung screening programme?’ (before I attend the lung screening appointment; at the same time as my screening appointment; with my screening results; 2–4 weeks after attending screening; 1–3 months after attending screening; more than 3 months after attending screening)*.

Five health behaviours were assessed. Fruit and vegetable consumption was measured using two items [24]: *‘Over the past month, how many portions of fruit / vegetables did you usually eat?’.* Participants who consumed five or more portions daily were categorised as meeting guidelines. Body Mass Index (BMI) was calculated from self-reported height and weight. Implausible BMI data were excluded (BMI < 14 / > 50). BMI was dichotomised to ≥25 (overweight) vs <25 (not overweight). Physical activity was assessed using one item: *‘In the past week on how many days have you done a total of* 30 min *or more of physical activity, which was enough to raise your breathing rate?’* [25,26]. Participants taking part in physical activity on five or more days per week were classified as meeting guidelines. Smoking status was assessed using two items*: ‘Do you smoke at all nowadays?’* and ‘*Did you stop smoking completely in the last five years?’.* Alcohol consumption was measured using the AUDIT-C questionnaire [27]. Participants scoring five or more were categorised as ‘increasing or higher risk drinkers’.

Cancer risk factor awareness was assessed using an 11-item scale [28]. Participants were shown 11 cancer risk factors (e.g. smoking, overweight) and were asked to select ‘*Which of the following, if any, do you personally think increase a person’s chances of developing cancer?’.* Participants were given a score out of 11.

### 2.4 Analyses

Descriptive analyses estimated willingness to receive information around the time of screening, the effect of information provision on screening uptake and preferences for information timing and topics. A McNemar’s test was used to investigate differences between interest in advice in general, and if results would require further investigation. Corrected Pearson Chi-squared analyses investigated differences in proportions of people willing to receive each topic of advice by current health behaviour. Two adjusted binary logistic regression models were used to identify determinants of interest in advice, and determinants of being put off attending screening if advice were delivered. Weights were applied to unadjusted analyses to account for response bias. Weights were calculated by Ipsos MORI using gender, age, social grade, region, working status, housing tenure and ethnicity. Analyses were conducted in Stata/SE 14.2.

## 3 Results

### 3.1 Sample characteristics

Of those interviewed, 685 were current smokers or recent quitters aged 50–75. Participants were excluded from analyses if they had received a previous cancer diagnosis (n = 46), or indicated they would not attend lung screening if invited (n = 174); six participants met both exclusion criteria leaving a final sample of 459 (Table 1). The mean age was 59.4 years. Half were male (51.4%), the majority were White (93.9%), and educated below degree level (83.9%). Most participants smoked (74.4%), were overweight (56.8%), and did not meet guidelines for fruit and vegetable consumption (63.1%) and physical activity (60.9%). One third of participants (32.2%) were identified as increasing or higher risk drinkers. Participants recognised an average of 3 out of 11 cancer risk factors.

**Table 1:** Weighted and unweighted sociodemographic characteristics of the sample.

	Unweighted (n = 479)		Weighted (n = 459)	
	n	%	n	%
**Gender**				
Men	246	51.4	236	51.4
Women	233	48.6	223	48.6

**Ethnicity**				
White	450	94.3	428	93.9
Non-White	27	5.7	28	6.1

**Education**				
Qualifications below degree level	399	83.3	385	83.9
Degree level or above	80	16.7	74	16.2

**Current health behaviour**				
**Smoking status**				
Current	352	73.5	341	74.4
Former	127	26.5	118	25.6

**Alcohol consumption (based on Audit-C score)**				
Increasing / higher risk drinker	326	31.7	147	32.2
Lower risk drinker	151	68.3	310	67.8

**Weight (based on BMI)**				
Overweight	195	56.4	187	56.8
Normal weight	151	43.6	143	43.2

**Diet (based on fruit and vegetable consumption)**				
Does not meet guidelines	296	61.9	289	63.1
Meets guidelines	182	38.1	169	36.9

**Physical activity**				
Does not meet guidelines	182	38.0	179	39.1
Meets guidelines	297	62.0	279	60.9

	**M**	**SD**	**M**	**SD**
**Age**	60.4	7.2	59.4	7.1

**Cancer risk factor awareness**	3.0	2.7	3.0	2.7
(out of a possible score of 11)				

### 3.2 Willingness to receive lifestyle advice at lung cancer screening

Two thirds (63.6%, n = 292) were willing to receive lifestyle advice at lung cancer screening. Non-White ethnicity and greater cancer risk factor awareness were associated with greater willingness to receive advice at this time (Table 2). Compared with 61.5% (n = 236) of White participants, 93.0% (n = 26) of non-White participants were willing to receive lifestyle advice at lung cancer screening, although confidence intervals were wide (OR 7.0, 95% CI 1.6–30.4, p = 0.009). Odds of interest in advice increased with each additional cancer risk factor identified (OR 1.10, 95% CI 1.0–1.2, p = 0.023). In a scenario where screening required further investigations, 83.1% (n = 381) indicated they would be willing to receive lifestyle advice at lung cancer screening. A greater proportion of people were willing to receive advice in this scenario, compared with during lung screening in general (McNemar’s Chi^2^ = 123.77, p < 0.001).

**Table 2:** Determinants of interest in lifestyle advice at lung cancer screening (n = 474, adjusted model).

	OR (95% CI)	p
**Gender**		
Men	Ref	
Women	0.77 (0.52–1.13)	0.183

**Ethnicity**		
White	Ref	–
Non-White	7.0 (1.6–30.4)	0.009

**Education**		
Degree level or above	Ref	–
Qualifications below degree level	0.96 (0.56–1.63)	0.872

**Age**	0.99 (0.97–1.02)	0.663

**Cancer risk factor awareness**	1.10 (1.01–1.19)	0.023

### 3.3 Impact of lifestyle advice on intention to have lung screening

Most lung screening intenders indicated the provision of lifestyle advice at screening would make no difference to their decision to attend (63.2%, n = 289), or would make them more willing to attend if lifestyle advice was provided (22.9%, n = 105). However, 13.9% (n = 64) of participants indicated they would be less willing to attend screening if lifestyle advice was provided. When we dichotomised the sample into those who would find lifestyle advice off-putting vs. those who would not, no determinants were found (Table 3).

**Table 3:** Determinants of being put off attending lung screening if lifestyle advice were provided (n = 472, adjusted model).

	OR (95% CI)	p
**Gender**		
Men	Ref	
Women	0.62 (0.36–1.07)	0.085

**Ethnicity**		
White	Ref	–
Non-White	0.92 (0.30–2.79)	0.882

**Education**		
Degree level or above	Ref	–
Qualifications below degree level	1.61 (0.84–3.11)	0.154

**Age**	0.97 (0.93–1.01)	0.128

**Cancer risk factor awareness**	0.95 (0.85–1.05)	0.311

### 3.4 Information preferences at lung cancer screening

The preferred timings for the delivery of lifestyle advice at lung cancer screening were at the screening appointment (37.7%, n = 108), with the screening results (30.5%, n = 87), and before the screening appointment (17.3%, n = 50). Few people thought advice should be provided at a later date (2–4 weeks after attending screening = 7.5%, n = 22; 1–3 months after attending screening = 5.2%, n = 15; more than three months after attending screening = 1.9%, n = 5).

**Fig. 1:**
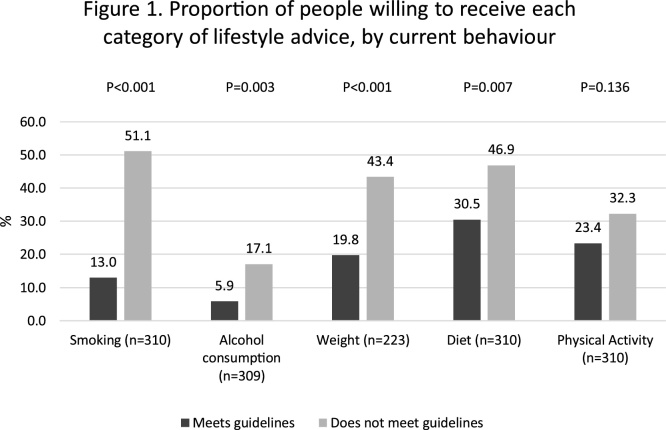
Proportion of people willing to receive each category of lifestyle advice, by current behavior.

Of the five topics of advice, interest was highest for smoking cessation (41.0%, n = 422, rising to 51.0% among current smokers) and dietary advice (40.5%, n = 118). One third of the sample were interested in advice about weight (32.4%, n = 94) and physical activity (29.0%, n = 84). One in ten participants (9.5%, n = 28) were interested in advice about alcohol consumption. Interest in advice about smoking, diet, weight and alcohol consumption was associated with adherence to the behaviour in question, with non-adherent people more interested in the relevant advice (Fig. 1).

## 4 Discussion

In this large, nationally representative study of people potentially eligible for lung cancer screening, two thirds were interested in receiving lifestyle advice in this context. Previous studies have evaluated smoking cessation advice at lung cancer screening [16], but this is the first study to determine interest in advice about other modifiable risk factors and to explore if this could affect screening attendance. Our results suggest lung screening attenders may be interested in several topics of advice. However, while the prospect of advice made some participants more willing to attend; a minority of participants reported the provision of lifestyle advice would make them less likely to attend lung screening.

Lung cancer screening uptake is low, particularly among current smokers and those from socioeconomically deprived populations [20,21]. Within our sample of people who intend to attend lung screening if invited, 14% indicated they would be less likely to participate if they knew lifestyle advice would be provided. While a minority, the potential impact that lifestyle advice may have on screening uptake for this group is important to understand, and so recommendations for future research are twofold. Firstly, future research should focus on identifying and characterising people who might be put off attending lung screening in a real world context, and their reasons for being deterred. We did not identify any sociodemographic determinants of being deterred, although sample sizes were small meaning associations may have been missed. Clearer insights into this group may be found by recruiting a larger sample, or by purposively sampling people who would be deterred by the provision of lifestyle advice at lung screening. Secondly, future research should focus on understanding how information and interventions can be delivered in a way that minimises any negative effects on screening uptake.

In keeping with previous research, greater cancer risk factor awareness was positively associated with interest in lifestyle advice at lung cancer screening [19,29]. While these data were from a cross-sectional sample, this finding suggests that increasing awareness could be a key aspect of engaging people with lifestyle advice within the cancer screening context. A greater proportion of non-White participants were interested in lifestyle advice at lung screening, which has previously been reported among non-White cervical cancer screening intenders [17]. However, only a small proportion of our sample were non-White highlighting the need for research with larger, more ethnically diverse samples. Qualitative research may be particularly useful tool for determining why non-White populations may be more receptive to advice in this setting, and for understanding the information needs of non-White lung screening attendees.

Our findings provide valuable information about preferences for advice delivered at lung screening. Most participants wanted to receive information close to the screening appointment (before attending, during the appointment or with results). It may be necessary to offer information at multiple stages to appeal to different preferences, to reinforce messages and to promote continuity of support for behaviour change. A greater proportion of our sample were willing to receive lifestyle advice if their results required further investigations. Increased smoking cessation rates have been observed among lung screening attendees with abnormal results, compared with attendees with normal screening results [30,31]. This suggests interventions may benefit from being tailored by screening result.

There is support for the implementation of smoking cessation interventions within the contest of lung cancer screening, and tentative support of the efficacy of these interventions [1,16]. However, research to date has not extended the focus of behavioural interventions at lung screening beyond smoking cessation. Our research suggests lung screening could be an opportunity to deliver lifestyle advice on various topics, including diet, weight and PA. Health behaviour was associated with interest in advice about smoking, alcohol consumption, diet and weight, which suggests a tailored, multiple risk factor approach may be appropriate. Interest in alcohol consumption advice was, however, low within our sample. This is a concern as a clustering of health behaviours means alcohol consumption is higher among smokers than non-smokers, and therefore likely to be higher among lung screening attendees [8,9]. It is important to consider how and when different types of advice could be offered to optimise their acceptability and effectiveness.

This research has limitations. Hypothetical scenarios were used to gauge interest in lifestyle advice among lung cancer screening intenders. An intention-behaviour gap has been observed for other cancer screening programmes [32,33], so it is likely that a proportion of our sample of screening intenders may not participate in lung screening if invited. As lung screening is not currently offered in England, the sample selected for this research may differ from the population invited to participate in lung screening if an organised programme is implemented in the future. Our criteria were based on the age-range used in a previous screening trial and smoking history, as it was not possible to calculate lung cancer risk [23]. Therefore, our sample may not represent the sample eligible for an eventual UK lung screening programme. Finally, the measures used within this study may limit our findings. The self-report measures used to assess health behaviour may be subject to response biases [19]. The choice and categorisation of sociodemographic covariates, such as the dichotomisation of ethnicity and the use of educational attainment as a marker of social position, may explain why no sociodemographic determinants of interest in advice were identified.

## 5 Conclusion

Lung cancer screening may offer an opportunity to provide advice about a range of behavioural cancer risk factors which may be prevalent among lung screening attendees. Future work should focus on developing effective and acceptable interventions in this setting, while ensuring strategies are aligned with those improving informed participation in lung screening.

## Conflict of interest statement

None declared.

## Funding

This research was supported by Cancer Research UK (C416/A19488, C1418/A14134, C7492/A17219, C44620/A23517) and Yorkshire Cancer Research (L389RB and L389SS).

## Role of the funding source

The funding sources did not contribute to the development of the study or preparation of the manuscript.
