# Supplementary material for: Interest in lifestyle advice at lung cancer screening: Determinants and preferences
Source: Lung Cancer. 2019 Feb;128:1–5. doi: 10.1016/j.lungcan.2018.11.036 (PMC6345624; doi:10.1016/j.lungcan.2018.11.036)
Supplement: Supplementary file 1 [file mmc1.docx]

**Supplementary File 1**

| **Variable** | **Measure/s** | **Response options** |
| --- | --- | --- |
| Age | What is your exact age? | N/A |
| Gender | Which of the following best describes how you think of yourself? | Male  Female  In another way |
| Ethnicity | Which group on this card do you consider you belong to? | (options presented on card)  White British  White Irish  White Gypsy/Traveller  White Other  Mixed White /Black Caribbean  Mixed White /Black African  Mixed White and Asian  Mixed other  Asian Indian  Asian Pakistani  Asian Bangladeshi  Asian Chinese  Asian other  Black African  Black Caribbean  Black other  Arab  Other |
| Educational attainment | Using this card, please tell me which, if any, is the highest educational or professional qualification you have obtained? | (options presented on card)  GCSE/O-Level/CSE  Vocational Qualifications (NVQ1+2)  A-Level or equivalent (NVQ3)  Bachelor degree or equivalent (NVQ4)  Masters/PhD or equivalent  Other  No formal qualifications  Still studying |
| Cancer risk factor awareness | Which of the following, if any, do you personally think increase a person’s chances of developing cancer?  Please select all that apply. | 1. Having a close relative with cancer 2. Getting sunburnt more than once as a child 3. Exposure to another person’s cigarette smoke 4. Doing less than 30 minutes of moderate physical activity 5 times a week 5. Being over 70 years’ old 6. Infection with HPV (Human Papillomavirus) 7. Smoking any cigarettes at all 8. Eating red or processed meat once a day or more 9. Eating less than 5 portions of fruit and vegetables a day 10. Drinking more than 1 unit of alcohol a day 11. Being overweight (having a BMI over 25) 12. None of these |
| Fruit and vegetable consumption | Over the past month, how many portions of fruit did you usually eat? Please include fruit eaten at meal times or as a snack.  Examples of a portion are:   - 1 apple or banana - A large slice of melon - 2 plums or satsumas - A small bowl of grapes - 2 tablespoons of tinned fruit - ½ tablespoon of dried fruit.   Over the past month, how many portions of vegetables did you usually eat? Please include vegetables eaten at meal times or as a snack.  Examples of a portion are:   - 2 heaped tablespoons of broccoli or carrots - 3 tablespoons of sweetcorn or peas - A bowl of salad | (presented separately for fruit and vegetable measures)   1. Less than 1 portion per week 2. 1 portion per week 3. 2-3 portions per week 4. 4-6 portions per week 5. 1 portion per day 6. 2 portions per day 7. 3 portions per day 8. 4 portions per day 9. 5 or more portions per day |
| Height and weight (for BMI) | What is your weight? Please give this as your weight when you are not wearing shoes.  What is your height? Please give this as your height when you are not wearing shoes. | (Three ways of answering provided)   1. Stones (st) and pounds (lbs) 2. Pounds (lbs) 3. Kilograms (kg)   (Two ways of answering provided)   1. Feet (ft) and inches 2. Centimetres (cm) |
| Physical activity | In the past week on how many days have you done a total of 30 minutes or more of physical activity, which was enough to raise your breathing rate?  This may include sport, exercise, and brisk walking or cycling for recreation or to get to and from places, but should not include housework or physical activity that may be part of your job. | 1. 1 day 2. 2 days 3. 3 days 4. 4 days 5. 5 days 6. 6 days 7. 7 days 8. None |
| Smoking | Do you smoke at all nowadays?  [For former smokers]  Did you stop smoking completely in the last five years? | 1. I smoke cigarettes (including hand-rolled) every day 2. I smoke cigarettes (including hand-rolled), but not every day 3. I do not smoke cigarettes at all, but I do smoke tobacco of some kind 4. I have stopped smoking completely in the last year 5. I stopped smoking completely more than a year ago 6. I have never been a smoker (i.e. smoked for a year or more)   Yes  No |
| Alcohol consumption (AUDIT-C) | How often do you have a drink containing alcohol?  How many units of alcohol do you drink on a typical day when you are drinking?  How often have you had 6 or more units if female, or 8 or more units if male, on a single occasion in the last year? | 1. Never (0 points) 2. Monthly or less (1 point) 3. 2 - 4 times per month (2 points) 4. 2 - 3 times per week (3 points) 5. 4+ times per week (4 points) 6. 0-2 (0 points) 7. 3-4 (1 point) 8. 5-6 (2 points) 9. 7-9 (3 points) 10. 10+ (4 points) 11. Never (0 points) 12. Less than monthly (1 point) 13. Monthly (2 points) 14. Weekly (3 points) 15. Daily or almost daily (4 points) |
| Intention to attend lung screening | A new screening test is being developed to find lung cancer at an early stage. It would use a type of x-ray called a chest CT scan. The scan takes pictures of the lungs which are then checked for the early signs of lung cancer. The screening test would be offered to people who smoke or used to smoke, not people going to their GP with symptoms of lung cancer.  If your GP invited you to have a lung cancer screening test as part of an NHS lung cancer screening programme, would you take up the offer? | 1. Yes, definitely 2. Yes, probably 3. No, probably not 4. No, definitely not |
| Willingness to receive lifestyle advice at lung screening | Would you be willing to receive advice about making healthy lifestyle changes (for example, diet or physical activity) as part of a lung cancer screening programme? | 1. Yes, definitely 2. Yes, probably 3. No, probably not 4. No, definitely not 5. Not sure |
| Willingness to receive lifestyle advice at lung screening if further investigations required | Would you be willing to receive lifestyle advice if your lung cancer screening result suggested you needed to have further investigations? | 1. Yes, definitely 2. Yes, probably 3. No, probably not 4. No, definitely not 5. Not sure |
| Impact of advice on lung screening uptake | If you knew you would receive advice about lifestyle (for example, diet or physical activity) as part of a lung cancer screening programme, would this affect your willingness to attend lung screening? | 1. Yes, I would be more willing to attend 2. Yes, I would be less willing to attend 3. No, it would not affect my willingness to attend |
| Interest in topics of advice | If you were to attend lung cancer screening in the future, which of the following, if any, would you be interested in receiving information or advice about?  Please select all that apply | 1. How to have a healthy diet 2. How to maintain a healthy weight 3. How to increase your physical activity 4. How to stop smoking 5. How to reduce your alcohol consumption 6. None of these |
| Preferred timing of advice | When would you prefer to receive lifestyle advice as part of a lung cancer screening programme? | 1. Before I attend the lung screening appointment 2. At the same time as the screening appointment 3. With my screening results (around two weeks after attending screening) 4. 2-4 weeks after attending screening 5. 1-3 months are attending screening 6. More than 3 months after attending screening |
